# Rapid Assessment of Non-Verbal Auditory Perception in Normal-Hearing Participants and Cochlear Implant Users

**DOI:** 10.3390/jcm10102093

**Published:** 2021-05-13

**Authors:** Agathe Pralus, Ruben Hermann, Fanny Cholvy, Pierre-Emmanuel Aguera, Annie Moulin, Pascal Barone, Nicolas Grimault, Eric Truy, Barbara Tillmann, Anne Caclin

**Affiliations:** 1 Lyon Neuroscience Research Center, CNRS, UMR5292, INSERM, U1028, F-69000 Lyon, France; ruben.hermann@chu-lyon.fr (R.H.); fanny.cholvy@gmail.com (F.C.); pe.aguera@inserm.fr (P.-E.A.); annie.moulin@inserm.fr (A.M.); nicolas.grimault@cnrs.fr (N.G.); eric.truy@chu-lyon.fr (E.T.); barbara.tillmann@cnrs.fr (B.T.); anne.caclin@inserm.fr (A.C.); 2 Claude Bernard Lyon 1 University, F-69000 Villeurbanne, France; 3 ENT Department, Hôpital Edouard Herriot, Hospices Civils de Lyon, F-69000 Lyon, France; 4 Brain & Cognition Research Center (CerCo), CNRS, UMR 5549, University of Toulouse Paul Sabatier, F-31062 Toulouse, France; Pascal.BARONE@cnrs.fr

**Keywords:** hearing loss, pitch perception, auditory scene analysis, prosody, audiovisual integration

## Abstract

In the case of hearing loss, cochlear implants (CI) allow for the restoration of hearing. Despite the advantages of CIs for speech perception, CI users still complain about their poor perception of their auditory environment. Aiming to assess non-verbal auditory perception in CI users, we developed five listening tests. These tests measure pitch change detection, pitch direction identification, pitch short-term memory, auditory stream segregation, and emotional prosody recognition, along with perceived intensity ratings. In order to test the potential benefit of visual cues for pitch processing, the three pitch tests included half of the trials with visual indications to perform the task. We tested 10 normal-hearing (NH) participants with material being presented as original and vocoded sounds, and 10 post-lingually deaf CI users. With the vocoded sounds, the NH participants had reduced scores for the detection of small pitch differences, and reduced emotion recognition and streaming abilities compared to the original sounds. Similarly, the CI users had deficits for small differences in the pitch change detection task and emotion recognition, as well as a decreased streaming capacity. Overall, this assessment allows for the rapid detection of specific patterns of non-verbal auditory perception deficits. The current findings also open new perspectives about how to enhance pitch perception capacities using visual cues.

## 1. Introduction

Auditory cognition allows us to perceive our environment and react properly to external stimuli. Communication via language is essential, but non-verbal auditory cognition also plays a primary role in the understanding of perceived stimuli, including prosody. Indeed, in speech communication, prosody perception is essential to understand the intentions and emotions of the speaker [1]. Moreover, non-verbal auditory abilities allow us to detect auditory signals in noise, and to better perceive and analyze the auditory scene [2]. Music perception also relies on our abilities to process and determine melody and harmony in non-verbal auditory signals [3].

Non-verbal auditory perception can be altered in the case of peripheral hearing loss [4,5,6]. When the hearing loss is profound, one or two cochlear implants can partially restore hearing capacities. However, auditory perception in cochlear implant (CI) users can remain impaired due to the limited frequency resolution of the implant [7,8]. Indeed, the technical constraints of an implant do not allow for a fine-grained decomposition of the auditory signal compared to the decomposition of a healthy cochlea [9]. As a result, CI users still have difficulties hearing speech in noise, understanding prosody and perceiving music, even though post-lingually deafened CI users have a strong desire to enjoy music again. These non-verbal auditory perception difficulties are assumed to be related to a pitch perception deficit resulting from the degraded auditory input [10]. In particular, as CIs do not always stimulate the apical part of the cochlea and only a frequency range around 150 to 8500 Hz is transmitted (depending on the CI device and settings), the poor perception of low frequencies could negatively impact the sound quality for CI users [10]. For instance, music and emotional prosody contain spectral energy outside this range [11,12]. In order to simulate the pitch perception deficit of CI users in normal-hearing (NH) listeners, a signal-processing tool referred to as a “vocoder” can be used [13]. Similarly to the CI, vocoders filter the sounds in frequency bands and extract the temporal envelope of the sound [14]. Consequently, the pitch discrimination thresholds measured in NH participants with vocoded sounds are higher than those in NH participants with original sounds (meaning non-vocoded sounds), showing similar pitch deficits to CI users [9,13,14]. Here, we aim to assess, using a short series of listening tests, the pattern of non-verbal auditory perception deficits in CI users and NH participants with vocoded sounds, compared to NH listeners with original sounds. We thus review below the documented deficits of non-verbal auditory perception in CI users.

### 1.1. Music Perception

Pitch perception has a key role in music processing: pitch discrimination is essential to perceive melody and harmony in music [14,15]. In CI users, deficits of music processing affect multiple dimensions. Musical features relating to pitch, such as timbre and harmonicity, are less well perceived by CI users than NH listeners [16,17,18,19]. Musical time processing was first thought to be relatively preserved in CI users [20,21,22]. However, when subjected to complex musical tasks (with pitch variations, not only sequences of tones), CI users do not perform as well on beat recognition and entrainment [22].

These deficits contribute to a general depreciation of music and a decreased quality of music listening in CI users [23], with a correlation between pitch perception abilities and music appreciation [24]. Regarding musical emotions, CI users showed a deficit for recognition compared to NH listeners, especially for sadness [16,25,26,27] or fear stimuli [28]. Some studies showed different arousal scores, but there were similar valence scores in CI users compared to NH listeners [8,25], suggesting that the musical emotion processing deficit is not a general deficit of music perception. Despite these limitations, some CI users still enjoy music, and have a strong desire to enhance this pleasure of music listening [29,30]. Overall, these results suggest that musical emotion recognition is possible in CI, but depends on features of the implantation as well as of the patient him/herself [31]. Music processing and appreciation might depend on the time of deafness and implantation: post-lingually deafened and implanted patients complain more about music depreciation compared to early-deafened late-implanted patients or prelingually implanted children [29]. Furthermore, some studies suggest that CI users might have implicit processing of pitch that could help them to enjoy music [32].

Overall, studies of music perception report that music appreciation remains poor in CI users [8,16,23,24,25,26,27,28,29]. Even if it might depend on previous musical knowledge [31], music perception—especially relating to pitch [24,33]—could possibly still be improved with CIs despite the technical limitations thanks to implicit knowledge related to pitch [29,32].

### 1.2. Prosody Perception

Even though speech in silent environments is quite well perceived by CI users, the pitch deficit still limits their non-verbal auditory perception in speech signals. For intentional prosody, CI users have demonstrated poor perception abilities [34,35] as well as a deficit for production [35]. This deficit was associated with difficulties in perceiving word and sentence stress [36]. For emotional prosody, CI users have deficits when visual cues are unavailable [28,37]. These difficulties are reflected in the electrophysiological correlates of emotional prosody processing, with a decreased N1-P2 in response to emotional bursts, reflecting an altered encoding of speech emotions in CI users [38]. However, it seems that this deficit could be partially compensated with enhanced musical exposure and training [39].

### 1.3. Auditory Scene Perception

Even when speech comprehension in a quiet environment might be quite effective, CI users still experience difficulties in hearing and understanding speech in noise [40,41,42]. Nevertheless, research investigating speech-in-noise perception has shown that a CI is helpful for enhancing speech perception in noisy situations [43].

As poor verbal working memory and lexical ability can limit speech perception in noise [44], tasks without speech were designed in order to better assess specific difficulties for hearing-in-noise, such as the Music-in-noise task developed for NH participants and musicians by Coffey et al. [45]. Hearing-in-noise perception mostly relies on the ability of the listener to separate auditory sources and to focus on the relevant source. Moreover, this ability seems not only to be associated with age-related hearing loss but also to depend on the cognitive abilities of participants, such as cognitive decline and dementia [46]. Streaming segregation tasks, initially developed for NH participants, allow us to determine how well listeners can discriminate between two auditory sources [47]. This segregation of auditory sources relies on the ability of the participant to spectrally separate these two sources [48]. Testing NH participants with vocoded sounds revealed that when the fundamental frequency (F0) discrimination becomes poorer, the segregation between two simultaneous sounds becomes very difficult [14,49], suggesting that this task could be a good index of CI users’ difficulties. Several studies investigating stream segregation in CI users revealed that they have increased perception of one stream, meaning that their abilities to distinguish several auditory sources might be reduced. Moreover, by using stream segregation tasks requiring the detection of rhythmic abnormalities, instead of the detection of frequency differences, as in classical stream segregation tasks [47,48,49,50], some studies measured indirectly the segregation abilities of CI users. These results showed that CI users seem not to experience automatic stream segregation [42,51,52,53,54]. CI users’ stream segregation performance is even worse when the background information is fluctuating, for example in a noisy environment [14]. Overall, the hearing-in-noise capacities of CI users remain a great challenge that needs to be addressed. Stream segregation tasks appear to be an efficient means of determining hearing-in-noise capacities in CI users, especially regarding the relevant pitch discrimination capacities [15].

### 1.4. Enhancing Pitch Perception with Visual Information

As reviewed above, non-verbal auditory perception is still a great challenge for CI users, and several studies have been conducted to improve this perception. It was suggested that cerebral plasticity could be induced in CI users (pre- and post-lingually) after implantation [7,55,56]. In particular, cross-modal plasticity in CI users could help to enhance their auditory capacities if correctly guided [7,57].

Indeed, in the perception of environmental stimuli, multisensory interactions are ubiquitous. For instance, the McGurk effect shows that the integration of visual and auditory information is essential for speech perception [58]. Several studies have shown the benefit of multisensory integration for perception rehabilitation [59,60,61]. Some findings suggest that multisensory integration is stronger when one of the sensory modalities is deficient. For example, in participants with reduced visual acuity, audiovisual interactions led to the improvement of visual detection thresholds beyond visual-only performance, which was not observed in control participants [62]. Similarly, in participants with a pitch processing deficit, such as in individuals with congenital amusia, visual stimulations helped them to improve their performance in an auditory pitch task [63]. Numerous studies have investigated audiovisual integration in CI users: the results using speech stimuli and faces showed enhanced auditory capacities in CI users thanks to audiovisual integration [64,65,66,67]. This audiovisual enhancement is more efficient in CI users compared to NH participants for all types of speech, even foreign-accented speech [68]. One study on non-linguistic speech processing, using voice gender identification, suggested that CI users are influenced more strongly by visual information than are NH participants, even for tasks not directly related to speech comprehension [69].

### 1.5. Rationale for the Present Study

Despite numerous studies investigating non-verbal abilities in CI users, few testing tools are available to assess these deficits. Testing batteries have been designed to detect auditory deficits in CI users, such as the Minimal Auditory Capabilities (MAC) [70] and the Minimum Speech Test Battery (MSTB) [71,72]. However, these tests mainly focus on speech intelligibility in quiet and noise, with only one subtest of the MAC testing for environmental sounds [70]. In comparison, the Basic Auditory Skills Evaluation Battery (BASE) [73] allows the detection of auditory deficits in CI users in a rather complete manner, including music processing. This battery is divided into twelve subtests of 3 to 5 min each that CI users can undertake to test their performance. However, the online implementation might create a difficult testing situation for some CI users. It is also noteworthy that all of these batteries require more than one hour of testing.

In the present study, we designed a new short series of listening tests to assess specifically certain aspects of non-verbal auditory perception in NH and CI users. This battery aimed to provide a rapid overview of non-verbal auditory capacities in an individual, and to be used for further auditory perception research on CI users and populations with hearing deficits, utilizing a quick assessment on a touchscreen tablet. This battery measures non-verbal auditory perception capacities with five tests: (1) pitch change detection [63], (2) pitch direction identification [74], (3) short-term memory for pitch [75], (4) stream segregation [50], and (5) emotional prosody [76]. Most of these tasks were selected on the basis of previous research investigating pitch processing in congenital amusia. Congenital amusia is a deficit in music perception and production that is associated with pitch perception [77] and pitch memory deficits [75,78,79,80,81]. These deficits have been traced down to an impaired fronto-temporal network which is involved in particular in pitch encoding [78,82,83]. Deficits in pitch change detection [84], pitch direction identification [85,86], and pitch short-term memory tasks [78,80,81] are hallmarks of the condition of congenital amusia, and we thus selected these three tasks as candidates to uncover the functioning of the combination of the peripheral auditory system and the cortical fronto-temporal network involved in pitch-related auditory cognition. Two further tests allow for the assessment of prosody perception and auditory scene analysis, as these two abilities are central in auditory non-verbal cognition and are altered in CI users, as is prosody perception in amusic participants [1,76,87,88,89]. Based on prior research with CI users on pitch and music processing, as well as on prosody and auditory scene analysis, we expected to observe deficits in CI users for all five tasks of the battery.

Finally, we also tested whether visual cues could improve the pitch processing of CI users, as well as the processing of full and vocoded signals in NH participants. We used informative and non-informative visual cues in non-verbal pitch perception and memory tasks (pitch change detection, pitch direction identification, and short-term memory) to test the potential usefulness of multisensory integration in these two populations. We hypothesized that CI users would have more difficulties than NH in general, but might benefit more strongly from the visual cues than do NH, even than NH in vocoded conditions, based on previous research on multisensory integration in CI users [64,66].

Overall, the present study presents a new set of five listening tests to assess important aspects of non-verbal auditory perception in NH participants and CI users. Its aim is to provide a rapid overview of the non-verbal auditory abilities of a given participant and, over the longer run, to provide a useful tool for further auditory research investigating various populations with hearing deficits.

## 2. Material and Methods

### 2.1. Participants

Ten cochlear implant users and ten normal-hearing participants were recruited (see Table 1 for demographical information). All of the control participants had normal hearing (hearing loss inferior to 20 dB at octave frequencies from 500 to 4000 Hz in both ears, measured with audiometry in silence, in accordance with the BIAP standards (Recommendation 02/1: Audiometric Classification of Hearing Impairments). There were six unilateral CI users with hearing-aids on the contralateral ear, and four bilateral CI users, all implanted after post-lingual deafness (see Table S1 for details about the CIs). We chose CI users with one year or more of implantation in order to have only CI users in the post-implantation chronic phase. Psychiatric and neuropsychological disorders were assessed by the Hospital where the CI users are medically taken care of. The participants were not diagnosed with psychiatric or neurological disorders, as assessed by a short questionnaire at inclusion in the study. The control participants were selected for their hearing-in-quiet capacities rather than their age, leading to age differences between the groups [20,69,90]. As age-difference could have played a role in the CI results, we performed Pearson correlations between each score obtained in the Results section and the age of the CI users, with Bonferroni correction for multiple testing. There was not any significant correlation between the age of the participants and their corresponding scores in all of the subtests (all pcorr > 0.4). We also ran a supplementary Bayesian Linear Regressions on the five tasks with age as a covariate, and no effect of age was significantly better than the null model for each task (all BF10 < 1.9). The study procedures were approved by an ethics committee (randomly selected at the national level, CPP Ile de France VI, ID RCD 2018-A02670-55), and all of the participants were paid for their participation and gave their written informed consent.

### 2.2. Listening Tests: Material and Procedure

**Figure 1:**
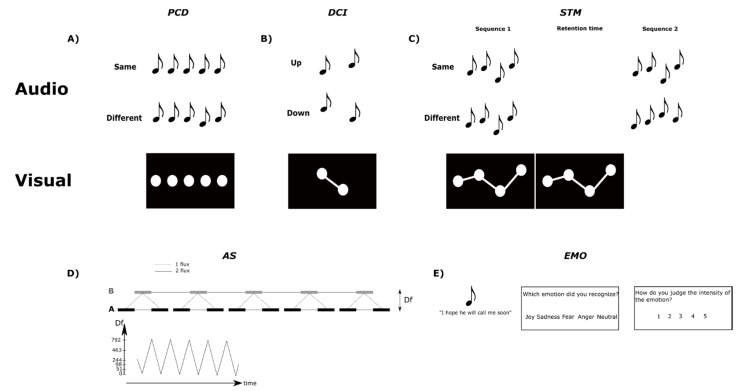
Schematics of the five listening tests of the battery. (**A**) In the PCD test, the participants have to determine whether the fourth note is identical to or different from the others. (**B**) In the DCI test, the participants have to determine if the second note is higher (“up”) or lower (“down”) than the first note. (**C**) In the STM test, the participants have to compare two melodies (sequence 1 and sequence 2) and determine if they are identical or different. The bottom panels of (**A**–**C**) present a visual representation of the tones played simultaneously with the visual information. Note that the visual stimuli (disks) appear one at a time, simultaneously with a tone, and remain on the screen during the rest of the stimulation (PCD, DCI, STM), as well as during the retention delay before S2 (STM). (**D**) In the AS test, the participants hear a sequence of notes with ABA triplet repetitions (see the schematic on the top row of the panel), the frequency of A is fixed, and the frequency of B changes across time (see the corresponding frequency difference, Df). The sequence can be perceived as one stream or two streams. (**E**) In the EMO test, the participants hear a sentence with one emotion, and have to choose the correct emotion (Joy, Sadness, Anger, Fear, or Neutral) and then rate the corresponding intensity of this emotion (except for Neutral stimuli).

Auditory non-verbal perception was assessed with five tests: pitch change detection (PCD), pitch direction change identification (DCI), pitch short-term memory (STM), auditory stream segregation (AS), and emotion recognition (EMO). All of the tests were implemented to run on an iPad touch tablet, allowing the participants to answer by touching large buttons presented on the screen (see Figure 1 for schematic representations of the five tests).

For the PCD, DCI and STM tasks, the same stimuli were used, with a roving of frequency across the trials. Note that we did not use intensity roving, either within or between the trials. We intended to measure how CI users could process frequency changes in stimuli as they can occur in everyday situations, and did not intend to assess which cues they used to do so: they might thus use some loudness cues [91,92] and not only pitch cues, in particular in the PCD and STM tasks. Furthermore, large intra-trial intensity roving creates a situation of interference which would be particularly deleterious in more cognitively demanding tasks such as the STM task. The stimuli for the PCD, DCI and STM tasks were synthetic harmonic tones (twelve harmonics), equalized in Root Mean Square (RMS) amplitude, each lasting 500 ms and presented with a within-sequence Inter-Stimulus-Interval (ISI) of 100 ms. The stimuli in the AS task were based on the same harmonic tones but with a duration of 100 ms. On half of the trials in the PCD, DCI and STM tasks, visual cues were presented (see Figure 1). They were composed of white disks with a diameter of 2 cm (100 px) on a black screen. For these three tests, auditory-only and audio-visual trials were presented in pseudo-random order, with no more than two repetitions of the same type of trial in a row. The visual stimuli were always informative regarding the timing of the audio stimulus (i.e., its onset). However, they provided task-relevant information about the tones’ pitch only for the STM and DCI tasks. In the DCI task, the visual stimuli indicated the correct answer for the task. The purpose of including audio-visual trials in the DCI task was not to gain any specific information, but rather for the sake of completeness, (1) to obtain visual information on half of the trials for the three pitch-based tasks, and (2) in anticipation of a training study, which is now ongoing, in which we are using informative, task-relevant visual information with the DCI task [74].

For the experiment with the NH participants, we also used vocoded sounds with 16, 8, and 4 channels (see below for details). The experiment took place in a quiet room. The participant was seated in front of the tablet with two loudspeakers (Logitech Z200) at a 70 cm distance from the participant’s head, with 40 cm between each speaker. We set the volume at 55 dB SPL for the NH participants and adjusted it to a comfortable listening level for the CI users (+/−5 dB SPL). The bilateral users performed the tests using both CIs. The unilateral users performed the tests using their CI and their hearing-aids in order to reproduce natural listening conditions in each CI user.

Each participant performed the five subtests in a random order, with each test presenting the stimuli in a pseudo-random order, with no more than two repetitions of the same type of stimulus in a row. For the NH participants, the order of the sound type (normal, vocoded four channels, vocoded eight channels, vocoded 16 channels) was also randomized, and all five tests for one sound type were presented in a row, in the same order for each sound type. Before each subtest, the participants received an oral explanation of the corresponding task, and the CI users also received written instructions to accompany the oral instructions. The participants performed a short training session in order to ensure that they understood the task, with the same range of frequencies as in the experimental trials. We checked the performance of this short training session for each participant before starting (at least 75% of correct responses). The entire session lasted about 30 min for CI users and two hours for NH participants. No response feedback was provided during the tests.

#### 2.2.1. Pitch Change Detection (PCD) Test

In one trial, the participants were presented with a sequence of five isochronous harmonic tones, all identical (standard harmonic tone) except for the fourth harmonic tone, which could differ in frequency (adapted from [63,84]). The standard frequencies were 165, 196, 262 or 392 Hz. The deviant frequencies were between 131 and 494 Hz, with the changes relative to the standard harmonic tone being between 1/16, 1/8, ¼, ½, 1, or 2 tones, either up or down compared to the standard. In total, 64 sequences were constructed with five notes. There were 16 identical trials (four trials for each standard) and 48 different trials (twelve trials per each standard, that is, one trial per deviant size, up and down). Non-informative visual cues were presented on half of the trials in addition to the harmonic tones (as in Albouy et al. [63]). Five circles appeared sequentially from left to right, synchronously with the harmonic tones. They were always positioned at the center of the vertical axis on the screen (see Figure 1), and hence were not informative as far as the pitch of the harmonic tone was concerned, meaning that they did not give sufficient information to perform the task, but gave information regarding the onset of the sound. The participants had to determine whether the fourth harmonic tone of the sequence was the same as or different from the other tones. After the end of the sequence, the participants had unlimited time to give their answer by tapping on either the “Same” or the “Different” button. After they had given their answer, the next trial was played automatically after an average delay of 1000 ms (700–1300 ms).

#### 2.2.2. Pitch Direction Change Identification (DCI) Test

The participants were presented with two harmonic tones at two different frequencies in each trial. The fundamental frequencies of the tones were between 123 and 523 Hz. The steps between the two harmonic tones could be of 0.5, 1, 1.5, 2, 2.5, 3, or 3.5 tones. In total, 56 two-tone sequences were constructed: 28 “up” sequences, with the second harmonic tone being higher in pitch than the first one, and 28 “down” sequences, with the second harmonic tone being lower in pitch than the first one. Informative visual cues were presented on half of the trials in addition to the harmonic tones. Two circles connected by a white bar appeared sequentially from left to right, and simultaneously with the onset of each harmonic tone. The vertical positions of the circles were centered on average on the two harmonic tones (to be at the center of the screen). Moreover, the vertical position of each circle was calculated according to the frequency of the corresponding harmonic tone: the higher the frequency, the higher the circle on the screen. In contrast to PCD, the visual cues were thus fully informative for pitch height (see Figure 1), but as they were only present in half of the trials, the participants were asked to base their judgements on their auditory perception. These visual cues aimed to reinforce the association between visual height and pitch [74], an effect we plan to exploit in future training experiments. The participants had to determine if the second harmonic tone was higher in pitch (Up) or lower (Down) than the first harmonic tone. After the end of the second harmonic tone, the participants had unlimited time to give their answer by tapping on either the “Up” or the “Down” button. After having given their answer, the next trial was played automatically after an average delay of 1000 ms (700–1300 ms).

#### 2.2.3. Pitch Short-Term Memory (STM) Test

The participants were presented with two melodies of four harmonic tones (S1 and S2), with S2 being either identical or different from S1 [75,93]. The fundamental frequencies of the harmonic tones were between 262 and 440 Hz (corresponding to notes between C4 and A4). In total, 32 melodies were constructed with four harmonic tones; each melody thus lasted 2300 ms. In total, there were 16 identical and 16 different trials. For the different trials, changes of one harmonic tone could occur on the second or third harmonic tone. The changes could be of 1.5, 2, 2.5, 3.5, or 4.5 tones, all entailing a change of contour. The delay between the two melodies of a trial was 1000 ms. Informative visual cues were presented on half of the trials in addition to the harmonic tones; they were presented during S1, and in the delay between S1 and S2. This allowed us to draw conclusions about the potential benefit of visual information during the encoding and maintenance phase of a memory task. The circles were connected by white bars, appearing consecutively and simultaneously with the onset of each harmonic tone of the first sequence. The circles’ vertical positions were centered on average over the four harmonic tones, and each vertical circle position was calculated according to the frequency of the corresponding harmonic tone: the higher the frequency, the higher the circle would be on the screen (see Figure 1). The participants had to determine if the second melody was the same or different from the first melody. After the end of the second melody, the participants had unlimited time to give their answer by tapping on “Same” or “Different” button. After they gave their answer, the next trial was played automatically after an average delay of 1000 ms (700–1300 ms).

#### 2.2.4. Auditory Stream Segregation (AS) Test

A sequence was constructed on the model ABA (with A being the standard harmonic tone and B being a harmonic tone with a varying frequency, both lasting 100 ms). The ISI between A and B was 20 ms, and the interval between two ABA triplets was 140 ms [47,50]. Five triplets were repeated for each frequency of B. The fundamental frequency of A was 196 Hz, and B was 196, 247, 294, 440, 659 or 988 Hz (i.e., ranging from 0 to 28 semitones with respect to 196 Hz). The sequence started with a fundamental frequency of B at 440, going down to 196 Hz, then up to 988 Hz, and down again to 196 Hz. This up-and-down pattern was repeated five times, terminating with B having a frequency of 294 Hz. In total, the AS sequence lasted approximately 2.5 min. During the sequence, the participants had to tell if they heard one stream (meaning they perceived the sequence as “integrated”) or two streams (meaning they perceived the sequence as “segregated”). They gave their answer by tapping on either the “1 stream” or the “2 streams” button. Once one button was selected, it remained selected until the participant changed their answer (the selected button remained highlighted). The participants could respond as many times as they wanted during the sequence.

#### 2.2.5. Emotion Recognition (EMO) Test

Twenty sentences were taken from Pralus and Fornoni et al. [76]. These sentences were semantically neutral in French: “J’espère qu’il va m’appeler bientôt” (“I hope he will call me soon”) and “L’avion est presque plein” (“The plane is almost full”). These sentences were uttered with different emotions by male and female actors. For each emotion (joy, neutral, sadness, anger, fear), four sentences were used, half pronounced by a male voice and half by a female voice. The stimuli lasted on average 1470 ms (+/−278 ms) and were equalized in RMS amplitude. In each trial, the participants listened to a stimulus and were asked to select the recognized emotion from five options (joy, neutral, sadness, anger, fear). After having given their response, they were asked to rate the intensity of the selected emotion from 1 (not intense) to 5 (very intense), except for the stimuli judged as neutral (as in Pralus and Fornoni et al., [76]). They had unlimited time to give their answer. After the intensity rating response, the following stimulus was played automatically after an average delay of 1000 ms (700–1300 ms).

#### 2.2.6. Vocoded Sounds

Three vocoded conditions simulating cochlear implants with different numbers of channels were created using MatLab R2016a (Mathworks, Inc, Natick, MA, USA). For the complete vocoding procedure, see Massida et al. [94] and Rouger et al. [64]. All of the sounds presented in the battery were analyzed through 4, 8, or 16 frequency bands using sixth-order IIR elliptical analysis filters. We extracted the temporal envelope by half-wave rectification for each of these frequency bands. The envelope was smoothed with a 500 Hz low-pass third order IIR elliptical filter. We used this extracted envelope to modulate a white noise given by a generator. The obtained signal was filtered with the same filters used previously for the frequency bands. We additively recombined the signals from each frequency band and adjusted the acoustic level obtained to match the original sound level based on RMS.

### 2.3. Data Analysis

A Bayesian approach was used to analyze the data [95,96]. We analyzed the data with Bayesian mixed repeated-measures analyses of variance (ANOVA), as implemented in the software JASP [97]. Bayesian analyses allow us to perform a model comparison and to select the best model (with the best evidence) given the data. In the first set of analyses, we investigated the effect of vocoding in NH participants’ data with Sound Type as a within-subject factor, with four levels (Non-vocoded, Vocoded with four, eight, or 16 channels). Other relevant factors depending on the task are detailed below. In the second set of analyses, we compared the groups (NH control data for non-vocoded sounds vs. CI), hence the between-participants factor Group was included for all of the tasks. We report the Bayes Factor (BF) as a relative measure of evidence. In order to interpret the strength of evidence (according to [95]), we considered a BF under three to be weak evidence, a BF between three and 10 to be positive evidence, a BF between 10 and 100 to be strong evidence, and a BF higher than 100 to be decisive evidence. BF10 indicates the evidence of H1 (a given model) compared to H0 (the null model), and BFinclusion indicates the evidence of one effect over all of the models. As no post-hoc tests with correction for multiple comparison have as yet been developed for Bayesian statistics [96,97], we used *t*-tests with Holm–Bonferroni correction for multiple comparisons.

For the PCD, DCI, and STM tests, we analyzed the percentage of correct responses with Modality (auditory or audiovisual) as a within-participant factor, and the factors Sound Type or Group (as described above). For PCD (different trials only) and DCI, we ran an additional analysis including the factor of Difficulty (different change sizes). For PCD, we also ran the analysis for identical trials only. For STM, we also analyzed the percentage of Hits (correct responses for different trials) minus the percentage of False Alarms (incorrect responses for the same trials) to correct for potential response bias.

For the AS test, we analyzed first the total time spent in the perception of one stream or two streams (thus excluding the time needed to give the first answer), and second, the mean frequency corresponding to changes in the number of streams perceived, with Sound Type or Group as a factor.

For the EMO test, we analyzed the percentages of correct responses and intensity ratings with Emotion (joy, sadness, anger, fear or neutral) as a within-participant factor, and the factors Sound Type or Group. Note that for the intensity ratings, the emotion factor had only four levels, as the neutral stimuli were not rated for intensity. We analyzed only the intensity ratings for trials with correctly recognized emotions (as in Pralus, Fornoni et al., 2019). The confusion matrices were calculated based on the percentage of responses given for each type of emotion compared to the expected emotion.

In addition, in order to better understand the relationships between the pitch tasks (PCD, DCI, and STM) with similar auditory stimuli, we performed a Bayesian ANOVA on accuracy for the auditory trials only, with Task as a within-participant factor, and with the between-participants factor Group (CI users, NH participants).

## 3. Results

### 3.1. PCD Test

#### 3.1.1. Normal-Hearing Participants and Vocoded Sounds

**Figure 2:**
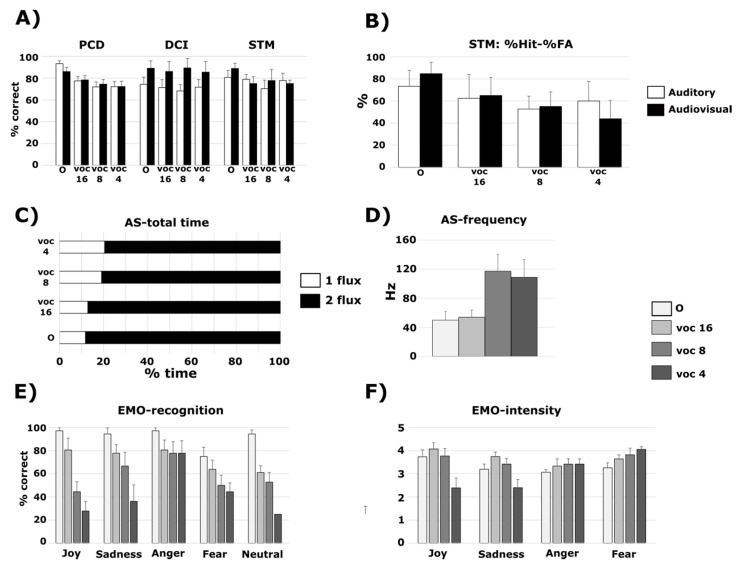
Results of the NH participants with original (O) and vocoded sounds (16, 8, or four channels) in the five tasks (PCD, DCI, STM, AS, and EMO). For PCD, DCI, and STM, the percentage of correct responses for the auditory (white bars) and audiovisual (black bars) trials are reported (**A**). For STM, an additional analysis of the percentage of Hits minus the percentage of False Alarm (FA) was performed (**B**). For AS, the total time (in percentage) in the perception of one (in white) or two (in black) streams is reported (**C**), as well as the mean frequency of the difference between tone A and Bat the change of perception (in Hertz) (**D**). For EMO, the percentage of correct recognition (**E**) and mean intensity ratings (**F**) are reported for each emotion. The error bars represent the standard error of the mean. PCD: Pitch Change Detection; DCI: Direction Change Identification; STM: Short-Term Memory; AS: Auditory Streaming; EMO: Emotional prosody.

After comparison to the null model, the best model showing positive evidence was the one with the main effect of Sound Type (BF10 = 4.09) (Figure 2A). The other models showed no noticeable evidence (BF10 < 1.2) (Table 2). This was confirmed by a decisive positive effect of Sound Type (BFinclusion = 3.15) only, while other specific effects showed no evidence (BFinclusion < 0.48). According to the t-tests with Holm–Bonferroni correction, the original sounds were significantly better recognized than the vocoded sounds with eight and four channels (all pcorr < 0.03).

**Figure 3:**
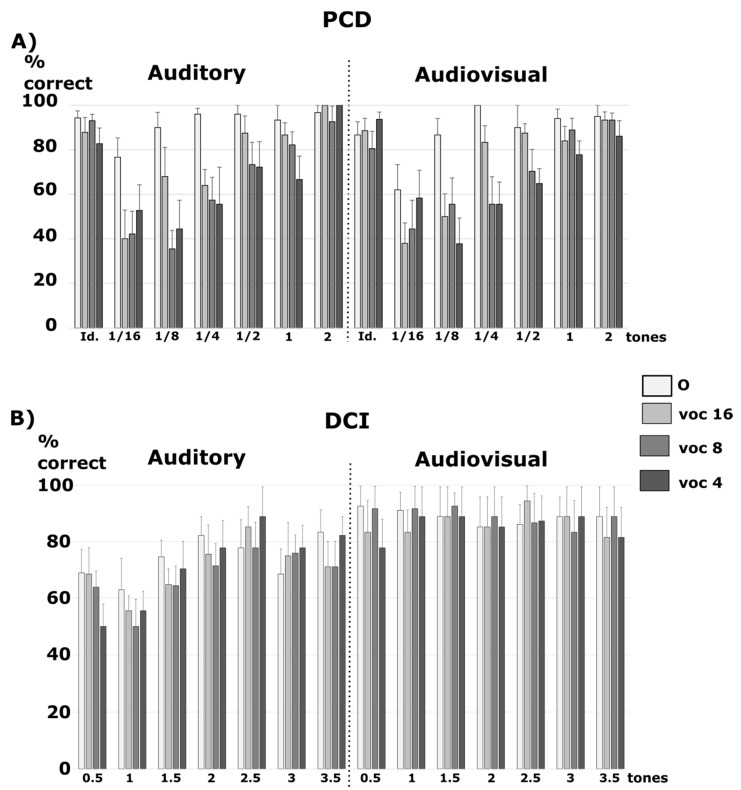
Results of the NH participants for original (O) and vocoded sounds (16, 8, and four channels) in the PCD (identical and different trials) and DCI, according to the size of the changes. The percentage of correct responses for auditory and audiovisual trials are reported for PCD (**A**) and DCI (**B**). For PCD, six size changes were used for the different trials: 1/16, 1/8, ¼, ½, 1 or 2 tones, and results for identical trials are also presented. For DCI, seven size changes were presented: 0.5, 1, 1.5, 2; 2.5, 3, or 3.5 tones. The error bars represent the standard error of the mean. PCD: Pitch Change Detection; DCI: Direction Change Identification; Id.: Identical trials.

In addition, we analyzed the percentage of correct responses for the different trials with the additional factor of Difficulty (six change sizes, see Methods) (Figure 3A). After comparison to the null model, the best model showing decisive evidence was the one with the main effects of Sound Type, Difficulty, and the interaction between the two (BF10 = 6.52 × 10^49^). It was 8.8 times better than the model with the main effects of Sound Type, Difficulty, Modality, and the interaction between Sound Type and Difficulty (BF10 = 7.37 × 10^48^), and 162 times better than the model with the main effects of Sound Type, Modality, Difficulty, the interaction between Sound Type and Modality, and the interaction between Sound Type and Difficulty (BF10 = 4.02 × 10^47^). All of the other models were at least 250 times less likely (BF10 < 2.6 × 10^47^). This was confirmed by a decisive specific effect of Difficulty (BFinclusion = 1.3 × 10^14^), Sound Type (BFinclusion = 1.3 × 10^14^), and the interaction between Sound Type and Difficulty (BFinclusion = 9.8 × 10^4^). Other specific effects showed no significant evidence (BFinclusion < 0.04). According to the t-tests with Holm–Bonferroni correction, the original sounds were significantly better recognized than the vocoded sounds with 16, eight, and four channels (all pcorr < 0.001). Over all of the types of sounds (original or vocoded), trials of difficulty of 1/16 and 1/8 tones led to poorer performance than all of the other trials (all pcorr < 0.017), trials of difficulty of 1/4 tone were less well performed than trials of difficulties of one and two tones (both pcorr < 0.009), and trials of difficulties of ½ and one tone were less well performed than trials of difficulty of two tones (both pcorr < 0.019). For the original sounds, trials of difficulty of 1/16 were less well performed than that with difficulties of ¼ and two (both pcorr < 0.038). For 16-channels vocoded sounds, trials of difficulties of 1/16 and 1/8 were less well performed than trials of difficulties of ½, one and two (all pcorr < 0.042); trials of difficulty of 1/16 were less well performed than that with a difficulty of ¼ (pcorr < 0.001). For eight-channel vocoded sounds, trials of difficulties of 1/16, 1/8 and 1/4 were less well performed than those of difficulties of one and two (all pcorr < 0.01), and trials of difficulties of 1/16 and 1/8 were less well performed than that of difficulty of 1/2 (both pcorr < 0.042). For four-channel vocoded sounds, trials of difficulties of 1/16, 1/8 and 1/4 were less well performed than that of difficulties of two (all pcorr < 0.001), and trials of difficulty of 1/8 were less well performed than that of difficulties of ½ and one (both pcorr < 0.025). Thus, overall, when fewer channels were used for the vocoded sounds, their discrimination became harder even with large physical differences.

For the identical trials, we also analyzed the percentage of correct responses (Figure 3A). None of the tested models explained the data better than the null model (BF10 < 1). However, there was a small specific effect of the interaction of Modality and Sound Type (BFinclusion = 2.5), but no other significant specific effects (BFinclusion < 0.7). None of the post-hoc tests were significant, and the largest numerical difference between the audio-only and audio-visual trials was for vocoded sounds with four channels.

#### 3.1.2. Cochlear Implant Listeners Compared to Normal-Hearing Participants

**Figure 4:**
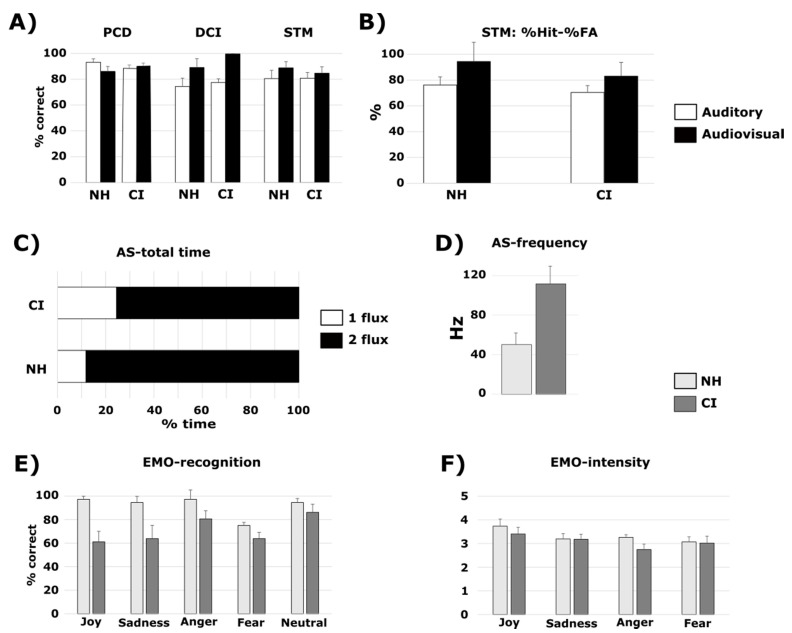
Results of the NH participants (with original sounds) and CI users in the five tasks (PCD, DCI, STM, AS, and EMO). For PCD, DCI and STM, the percentage of correct responses for the auditory (white bars) and audiovisual (black bars) trials are reported (**A**). For STM, an additional analysis of the percentage of Hits minus the percentage of False Alarms (FA) was performed (**B**). For AS, the total time (in percentage) in the perception of 1 (in white) or 2 (in black) streams is reported (**C**), as well as the mean frequency at change of perception (in Hertz) of the difference between tone A and B (**D**). For EMO, the percentage of correct recognition (**E**) and mean intensity ratings (**F**) are reported for each emotion. The error bars represent the standard error of the mean. PCD: Pitch Change Detection; DCI: Direction Change Identification; STM: Short-Term Memory; AS: Auditory Streaming; EMO: Emotional prosody.

The null model and the model with Modality, Group, and the interaction between the two factors (BF10 = 0.9) explained almost equally well the data, with the null model being only 1.1 times more likely to explain the data than the model with all of the effects and interactions (Table 2, Figure 4A). This was confirmed by only a small specific effect of the interaction between Modality and Group (BFinclusion = 1.37), and no other significant specific effects (BFinclusion < 0.9). The data pattern was clearer with separated analyses for the same and different trials, as seen below.

**Figure 5:**
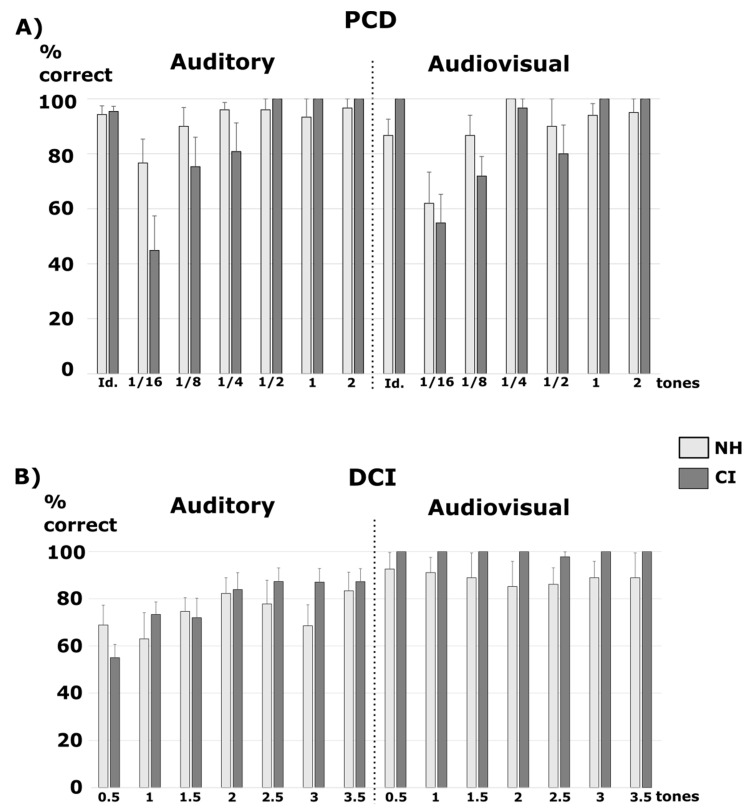
Results of the NH participants and CI users in the PCD and DCI, according to the size of the changes. The percentages of correct responses for auditory and audiovisual trials are reported for PCD (**A**) and DCI (**B**). For PCD, six size changes were present for the different trials: 1/16, 1/8, ¼, ½, 1, or 2 tones, and the results for identical trials are also presented. For DCI, seven size changes were presented: 0.5, 1, 1.5, 2; 2.5, 3, or 3.5 tones. The error bars represent the standard error of the mean. PCD: Pitch change Detection; DCI: Direction Change Identification; Id.: Identical trials.

In addition, we analyzed the percentage of correct responses for the different trials with the additional factor of Difficulty (six change sizes) (Figure 5A). After comparison to the null model, the best model showing decisive evidence was the one with the main effects of Group, Difficulty, and the interaction between the two (BF10 = 1.24 × 10^16^). It was 1.6 times better than the model with the main effect of Difficulty (BF10 = 7.68 × 10^15^), 3.4 times better than the model with the main effects of Group and Difficulty (BF10 = 3.61 × 10^15^), and 5.8 times better than the model with the main effects of Group, Difficulty, Modality and the interaction between Group and Difficulty (BF10 = 2.14 × 10^15^). All of the other models were at least 10 times less likely (BF10 < 1.2 × 10^15^). This was confirmed by a decisive specific effect of Difficulty (BFinclusion = ∞) and a weak specific effect of the interaction between Group and Difficulty (BFinclusion = 2.5). The other specific effects showed no significant evidence (BFinclusion < 0.8). According to t-tests with Holm–Bonferroni correction, over the two groups, trials of difficulty of 1/16 tone were less well categorized than all of other trials (all pcorr < 0.004), trials of difficulty of 1/8 tone were less well categorized than trials of difficulties of 1 and 2 tones (both pcorr < 0.012). In CI users, we found the same pattern of responses: trials of difficulties 1/16 and 1/8 were less well categorized than other difficulties (all pcorr < 0.025). In NH participants, only trials of difficulty of 1/16 tone were less well categorized than trials of difficulty of ¼, ½, 1 and 2 tones (all pcorr < 0.026).

We also analyzed the percentage of correct responses for identical trials (Figure 3A). The null model and the model with Modality, Group, and the interaction between the two factors (BF10 = 1.6) explained almost equally well the data, with the null model being only 1.6 times less likely to explain the data than the model with all of the effects and interactions (Table 2). This was confirmed by a small specific effect of the interaction between Modality and Group (BFinclusion = 2.3), and Group (BFinclusion = 1.5) and no other significant specific effects (BFinclusion < 0.8). According to the t-tests with Holm–Bonferroni correction, for audiovisual trials, CI users tended to have enhanced scores compared to NH listeners (pcorr = 0.07).

### 3.2. DCI Test

#### 3.2.1. Normal-Hearing Participants and Vocoded Sounds

After comparison to the null model, the best model showing decisive evidence was the one with the main effects of Modality (BF10 = 2.78 × 10^6^) (Figure 2A). It was 8.9 times better than the model with the main effect of Modality and Sound Type (BF10 = 3.12 × 10^5^), and 43 times better than the model with the two main effects of Sound Type and Modality, and their interaction (BF10 = 6.42 × 10^4^). The model with the main effect of Sound Type only showed no significant evidence (BF10 = 0.1) (Table 2). This was confirmed by a decisive specific effect of Modality (BFinclusion = 1.92 × 10^6^) only. The other specific effects showed no significant evidence (BFinclusion < 0.09).

In addition, we analyzed the percentage of correct responses with the additional factor of Difficulty (seven change sizes, see Methods) (Figure 3B). After comparison to the null model, the best model showing decisive evidence was the one with the main effects of Modality, Difficulty, and the interaction between the two (BF10 = 6.36 × 10^23^). It was 41.3 times better than the model with the main effects of Modality, Sound Type, Difficulty, and the interaction between Modality and Difficulty (BF10 = 1.54 × 10^22^), and 468 times better than the model with the main effects of Modality, Sound Type, Difficulty, and the interaction between Sound Type and Modality, and between Modality and Difficulty (BF10 = 1.36 × 10^21^). All of the other models were at least 1870 times less likely (BF10 < 3.4 × 10^20^). This was confirmed by the decisive specific effects of Modality (BFinclusion = 4.6 × 10^14^), Difficulty (BFinclusion = 3.8 × 10^4^), and the interaction between Modality and Difficulty (BFinclusion = 4020). Other specific effects showed no significant evidence (BFinclusion < 0.01). According to the t-tests with Holm–Bonferroni correction, trials of difficulties of 0.5 and 1 tone were less well categorized than trials of difficulties of 2.5, 3 and 3.5 tones (all pcorr < 0.05). Audiovisual trials were specifically better categorized than auditory trials for difficulty levels of 0.5, 1, 1.5 and 3 tones (all pcorr < 0.001).

#### 3.2.2. Cochlear Implant Listeners Compared to Normal-Hearing Participants

After the comparison to the null model, the best model showing decisive evidence was the one with the main effect of Modality (BF10 = 4178.2) (Figure 4A). It was 1.5 times better than the model with the main effects of Modality and Group (BF10 = 2708.5), and two times better than the model with the two main effects of Group and Modality, and their interaction (BF10 = 2113.5). The model with the main effect of Group only showed no significant evidence (BF10 = 0.5) (Table 3). This was confirmed by a decisive specific effect of Modality (BFinclusion = 3952.3) only. The other specific effects showed no significant evidence (BFinclusion < 1.2). As the three first models were very close, we report the post-hoc comparisons for the interaction. According to the t-tests with Holm–Bonferroni correction, both groups had better scores in the audiovisual trials compared to the auditory trials (pcorr < 0.001 for CI users and pcorr = 0.013 for NH). The CI users were significantly better in the audiovisual trials compared to the NH participants in the auditory trials (pcorr = 0.007), but not when comparing their performance in the auditory trials to the NH’s performance in the auditory trials. Note that on average across the audiovisual and auditory trials, the CI users thus performed better than the NH participants.

In addition, we analyzed the percentage of correct responses with the factor of Difficulty (seven change sizes, see Methods) (Figure 5B). After comparison to the null model, the best model showing decisive evidence was the one with the main effects of Modality and Difficulty, and the interaction between the two (BF10 = 2.28 × 10^13^). It was 1.52 times better than the model with the main effect of Modality (BF10 = 1.5 × 10^13^); 1.8 times better than the model with the main effects of Modality, Group, Difficulty, the interaction between Group and Modality, and between Modality and Difficulty (BF10 = 1.26 × 10^13^); and 1.9 times better than the model with the main effects of Modality, Group, Difficulty, and the interaction between Modality and Difficulty (BF10 = 1.19 × 10^13^). All of the other models were at least 2.7 times less likely (BF10 < 8.4 × 10^12^). This was confirmed by a decisive specific effect of Modality (BFinclusion = 1.87 × 10^13^) and a small positive effect of the interaction between Modality and Difficulty (BFinclusion = 3). The other specific effects showed no significant evidence (BFinclusion < 0.77). According to the t-tests with Holm–Bonferroni correction, the trials of difficulty of 0.5 tone had a tendency to be less well categorized than the trials of difficulties of 2.5 tones (pcorr = 0.14). The audiovisual trials were specifically better categorized than the auditory trials for difficulty levels of 0.5, 1 and 1.5 (all pcorr < 0.013).

### 3.3. STM Test

#### 3.3.1. Normal-Hearing Participants and Vocoded Sounds

None of the tested models explained the data (percentage of correct responses) better than the null model (BF10 < 0.5) (Table 2, Figure 2A). This was confirmed by there being no significant specific effects (BFinclusion < 0.4).

In addition, we analyzed the percentage of Hits minus the percentage of false alarms in NH participants with all sound types, in audio or audiovisual conditions (Figure 2B). None of the tested models explained the data better than the null model (BF10 < 0.6). This was confirmed by there being no significant specific effects (BFinclusion < 0.5).

#### 3.3.2. Cochlear Implant Listeners Compared to Normal-Hearing Participants

The null model and the model with Modality (BF10 = 1.7) explained the data almost equally well, with the null model being only 1.7 times less likely to explain the data than the model with all of the effects and interactions (Table 3, Figure 4A). This was confirmed by a small specific effect of Modality (BFinclusion = 1.3), and no other significant specific effects (BFinclusion < 0.4).

For the analysis of the percentage of Hits minus the percentage of False Alarms (Figure 4B), after comparison to the null model, the best model showing positive evidence was the one with the main effect of Modality (BF10 = 3). The other models showed no significant evidence (BF10 < 1.5). This was confirmed by the small evidence of Modality only (BFinclusion = 2.2), and the other specific effects showed no evidence (BFinclusion < 0.5). The performance in the audiovisual trials was better than performance in the auditory trials in both groups.

### 3.4. AS Test

#### 3.4.1. Normal-Hearing Participants and Vocoded Sounds

*Total time spent in one or two stream percepts* (Figure 2C). After comparison to the null model, the best model showing decisive evidence was the one with the main effects of Perception (one or two streams) and Sound Type, and the interaction between the two (BF10 = 3.28 × 10^48^). This model was 7.2 times more likely than the model with the main effect of Perception (BF10 = 4.3 × 10^47^), and 94 times more likely than the model with the two main effects of Perception and Sound Type (BF10 = 3.48 × 10^46^). The model with the main effect of Sound Type showed no evidence (BF10 = 0.07) (Table 2). This was confirmed by a decisive specific effect of Perception (BFinclusion = 9.8 × 10^13^), a strong specific effect of the interaction between Perception and Sound Type (BFinclusion = 28), and a positive specific effect of Sound Type (BFinclusion = 5.1). According to t-tests with Holm–Bonferroni correction, less time was spent in the perception of one stream compared to two streams for all types of sounds (all pcorr < 0.001). There was a tendency to spend less time in the perception of one stream with four-channel vocoded sounds compared to original sounds (pcorr = 0.069), and a tendency to spend more time in the perception of two streams with four-channel vocoded sounds compared to 16-channel vocoded sounds (pcorr = 0.069).

*Mean frequency at the change of perception* (Figure 2D). After comparison to the null model, the model with the main effects of Sound Type showed strong evidence (BF10 = 12.85) (Table 2). According to the t-tests with Holm–Bonferroni correction, there was a tendency for a higher mean frequency with eight-channel vocoded sounds compared to 16-channel vocoded sounds (pcorr = 0.1).

#### 3.4.2. Cochlear Implant Listeners Compared to Normal-Hearing Participants

*Total time spent in one or two streams percepts* (Figure 4C). After comparison to the null model, the best model showing decisive evidence was the one with the main effects of Perception (one or two streams) and Group, and the interaction between the two (BF10 = 6.4 × 10^16^). This model was 9.7 times more likely than the model with the main effect of Perception (BF10 = 6.6 × 10^15^), and 27.8 times more likely than the model with the two main effects of Perception and Group (BF10 = 2.4 × 10^15^). The model with only the main effect of Group showed no evidence (BF10 = 0.3) (Table 3). This was confirmed by a decisive specific effect of Perception (BFinclusion = ∞), a strong specific effect on the interaction between Perception and Group (BFinclusion = 28.8), and a positive specific effect of Group (BFinclusion = 6.8). According to the t-tests with Holm–Bonferroni correction, both groups spent less time in the perception of one stream compared to two streams (both pcorr < 0.001). The CI users spent significantly less time in the perception of two streams compared to the NH participants (pcorr = 0.034), and more time in the perception of one stream compared to the NH participants (pcorr = 0.024).

*Mean frequency at the change of perception* (Figure 4D). After comparison to the null model, the model with the main effect of Group showed positive evidence (BF10 = 5.1) (Table 3). The frequency difference between the A and B sounds at which switches between perceptions occurred was higher for the CI users’ group (111.6 Hz) than the NH participants’ group (49.8 Hz).

### 3.5. EMO Test

#### 3.5.1. Normal-Hearing Participants and Vocoded Sounds

*Emotion categorization* (Figure 2E). After comparison to the null model, the best model showing decisive evidence was the one with the two main effects of Sound Type and Emotion, and their interaction (BF10 = 5.68 × 10^19^). This model was 7.8 times more likely than the model with the two main effects of Sound Type and Emotion (BF10 = 7.3 × 10^18^), and 14,200 times more likely than the model with the main effect of Sound Type (BF10 = 4 × 10^15^). The model with the main effects of Emotion showed strong evidence (BF10 = 19.9) (Table 2). This was confirmed by a decisive specific effect of Emotion (BFinclusion = 1.06 × 10^4^) and Sound Type (BFinclusion = 3.3 × 10^14^), and a strong specific effect of the interaction between Emotion and Sound Type (BFinclusion = 31.1). According to the t-tests with Holm–Bonferroni correction, Anger was significantly better recognized than Joy and Neutrality (pcorr = 0.019 and 0.008 respectively). The original sounds were significantly better recognized than the vocoded sounds with 16, eight and four channels (all pcorr < 0.003). The vocoded sounds with 16 channels were significantly better recognized than the ones with eight and four channels (both pcorr < 0.001), and vocoded sounds with eight channels were better recognized than the ones with four channels (pcorr = 0.016). The original and vocoded sounds with 16 channels were significantly better recognized than the vocoded sounds with four channels for Joy, Sadness and Neutrality (all pcorr < 0.003). The original sounds and 16-channel vocoded sounds were significantly better recognized than the eight-channels vocoded sounds for Joy (pcorr = 0.023). The original sounds were significantly better recognized than the eight-channel vocoded sounds for Neutrality (pcorr = 0.011).

The confusion matrices (Table S2) showed that with vocoded sounds, fear was often confused with anger, which was never the case with the original sounds; indeed, with the original sounds, fear was more confused with sadness. Moreover, with the vocoded sounds, sadness was often confused with neutrality, whereas this confusion was not present with the original sounds.

*Intensity ratings* (Figure 2F). After comparison to the null model, the best model showing decisive evidence was the one with the two main effects of Sound Type and Emotion, and their interaction (BF10 = 2.8 × 10^5^). This model was 1202 times more likely than the model with the two main effects of Sound Type and Emotion (BF10 = 233.6), and 7777 times more likely than the model with the main effect of Sound Type (BF10 = 36.2). The model with the main effect of Emotion showed small positive evidence (BF10 = 3.7) (Table 2). This was confirmed by a decisive specific effect of Emotion (BFinclusion = 4973), Sound Type (BFinclusion = 3.4 × 10^4^), and the interaction between Emotion and Sound Type (BFinclusion = 4038). According to the t-tests with Holm–Bonferroni correction, Anger was rated as being significantly more intense than Fear and Sadness (pcorr = 0.04 and 0.004 respectively). Joy was rated as being more intense than Sadness (pcorr = 0.005). Vocoded sounds with 16-channels tended to be rated higher than four-channel vocoded sounds (pcorr = 0.078). Vocoded sounds with 16 channels were rated significantly higher than vocoded sounds with four channels for Joy and Sadness (both pcorr < 0.001).

#### 3.5.2. Cochlear Implant Listeners Compared to Normal-Hearing Participants

*Emotion categorization* (Figure 4E). After comparison to the null model, the best model showing decisive evidence was the one with the two main effects of Group and Emotion, and their interaction (BF10 = 4107.7). This model was seven times more likely than the model with the two main effects of Group and Emotion (BF10 = 588.8), and 60 times more likely than the model with the main effect of Emotion (BF10 = 68). The model with the main effect of Group showed positive evidence (BF10 = 8.7) (Table 3). This was confirmed by a decisive specific effect of Emotion (BFinclusion = 325.9), a strong specific effect of Group (BFinclusion = 45.5), and of the interaction between Emotion and Group (BFinclusion = 24.7). According to the t-tests with Holm–Bonferroni correction, Fear was significantly less recognized than Anger and Neutrality (all pcorr < 0.001). CI had lower recognition scores compared to NH for Joy (t(8) = 4.3 pcorr = 0.004) and for Sadness (t(8) = 3.5 pcorr = 0.038) (other pcorr > 0.8).

The confusion matrices (Table S3) showed that in CI users, joy was often confused with sadness, which was never the case for the control participants. Moreover, in the CI users, sadness was often confused with neutrality; this confusion was not observed for NH participants.

*Intensity ratings* (Figure 4F). After comparison to the null model, all of the models showed no significant evidence (BF10 < 1.1) (Table 3). This was confirmed by there being no significant specific effects (BFinclusion < 0.8).

Overall, the CI users showed prominent deficits in three out of the five listening tasks compared to the NH participants: Pitch Change Detection, Auditory Stream segregation and Emotional prosody recognition. In contrast, the CI users reached similar performance levels for the pitch Direction Change Identification task, as well as the pitch sequence Short-Term Memory task in comparison to the NH participants.

The NH participants with vocoded sounds showed deficits in three out of the five listening tasks: Pitch Change Detection, Auditory Stream segregation and Emotional prosody recognition (and their associated intensity ratings). These deficits partly correlated to the number of channels of the vocoder used.

In order to assess the potential differences between the unilateral and bilateral CI users, we compared the results of the two subgroups (unilateral vs bilateral CI users, see Table S4 for the individual results). No significant difference was found between the two groups for any of the tasks (all BF10 < 3 for models with the Group effect).

### 3.6. Relationships between the Tasks: Cochlear Implant Listeners Compared to Normal-Hearing Participants in Pitch Tasks (PCD, DCI and STM)

After comparison to the null model, the best model showing positive evidence was the one with the main effect of Task (BF10 = 7.25) (Figure S1). It was 1.3 times better than the model with the two main effects of Group and Task, and their interaction (BF10 = 5.45), and 2.1 times better than the model with the two main effects of Group and Task (BF10 = 3.42). The model with the main effect of Group was not significant (BF10 = 0.42). This was confirmed by a positive specific effect of Task (BFinclusion = 7.55), and a small specific effect of the interaction between Task and Group (BFinclusion = 1.8), and no other significant specific effect (BFinclusion < 0.8). According to the t-tests with Holm–Bonferroni correction, PCD showed greater recognition scores than DCI (pcorr = 0.018) and STM (pcorr = 0.018). In the control group, the PCD task showed greater recognition scores than the DCI (pcorr = 0.005). This was not the case in the CI users, reflecting the more homogenous results across the tasks in this group, with higher scores in DCI and lower scores in the PCD compared to the NH participants (see Figure S1).

## 4. Discussion

Overall, our study demonstrated that the rapid assessment of non-verbal auditory perception can be performed for NH participants and CI users. The CI users showed prominent deficits in three out of the five listening tasks compared to the NH participants: Pitch Change Detection, Auditory Stream segregation and Emotion (prosody) recognition. In contrast, they reached similar performance levels for the pitch Direction Change Identification task, as well as the pitch sequence Short-Term Memory task in comparison to the NH participants. This pattern of perceptual deficits of CI users was mostly mimicked in the NH participants with vocoded sounds, with a deficit partly correlated to the number of channels of the vocoder used. Both groups seemed to benefit from visual cues in the pitch tasks, but these effects were small and did not differ between the participant groups.

### 4.1. Patterns of Non-verbal Auditory Perception Deficits in CI Users and in NH Participants Hearing Vocoded Sounds

In the PCD test, which was the most basic task related to pitch perception in the battery, the CI users demonstrated a deficit compared to the NH participants for the trials with the smallest pitch differences. The CI users’ performance level was comparable to the performance level of the NH participants listening to vocoded sounds (Figure 2A and Figure 3A). These deficits were more pronounced for the difficult trials: the smaller the size change was, the bigger the deficits of the CI users and NH participants with vocoded sounds. These results were expected because CI and vocoded sounds only give a partial and degraded information about the pitch of the sound [7,8]. As we used roving pitches in the PCD task, we prevented frequency-related training and showed that the deficit was not specific to one frequency in particular.

In the DCI task, the participants did not need to recognize the pitch per se, but rather needed to distinguish a pitch difference and to infer a direction on this difference. The CI users showed no deficit on this task compared to the NH participants who listened either to the original sounds or the vocoded sounds. This could be linked to the fact that, compared to the PCD task, the participants were not asked to detect a pitch change, but rather to make a decision on a relationship between two tones, recognizing the contour. Previous work has demonstrated that CI users had remaining implicit pitch processing [32], which could have helped in this task. Moreover, in the DCI task, the pitch change sizes were bigger than in the PCD task. However, this task was not easier than the PCD. Indeed, for the NH participants, it was more difficult to correctly identify the direction of a pitch change (DCI) than to detect a pitch change (PCD).

As pitch is essential for music perception, and CI users often complain about their poor musical appreciation [23,24,28], we used a short music-like task to test their perception of melody information in the context of a short-term memory (STM) task. Surprisingly, the CI users did not show a deficit on this task, and performed similarly to NH participants with original sounds. Note that the NH participants did not show a deficit on this task with vocoded sounds either. Even though hearing loss can decrease cognitive abilities [98], various studies have demonstrated that compensating this hearing loss, in particular with CI, can improve cognitive performance [98,99,100,101]. The observed data pattern here suggests that the short-term memory abilities of CI users were sufficient to perform well at this memory task, even though the difference of melodies was based on a change on the pitch dimension, an acoustic dimension which is difficult to process for them. Moreover, as the to-be-detected difference included a change of contour, this feature could have helped CI users to detect this difference, similarly to the DCI task. Indeed, previous work on melodic contour identification (MCI task) revealed that CI users are able to use melodic contour information [102,103,104]. Their recognition scores are similar to NH participants when the number of semitones between notes is large enough [102], and they improve with the number of years of musical experience [105]. Overall, it appears that when the task is not purely pitch-perception centered, CI users can perform as well as NH participants. This could explain why, despite their poor pitch discrimination abilities, CI users are able to listen to and enjoy music [29,104]. It might be argued that the patients only succeeded because they were not impaired severely enough. However, the CI users tested here experienced difficulties with the PCD task and the two tasks AS and EMO, which are both more related to their everyday life perception.

In agreement with previous reports [42,51,52,53,54], our AS task detected the deficit of streaming segregation in CI users. Indeed, the CI users spent more time in the perception of one stream, meaning that their segregation was not performed as efficiently as that of the NH participants. This was confirmed by an increase of the frequency at the change of perception. Interestingly, these results were also found in NH participants with vocoded sounds, but only for the 4-channels vocoded sounds, i.e., the most degraded sounds. This reflects the deficit of CI users in everyday life in the segregation of two auditory sources [14,52,53], and more generally to hear signals in noise. However, both NH listeners and CI users spend considerable amounts of time in noisy situations, mostly trying to understand sounds in background noise [106]. In order to reflect this deficit, streaming segregation tasks have often been used, as they reflect a way of understanding hearing-in-noise apart from speech comprehension per se. Some studies suggest that these tasks only show a need for more time to make a decision and subjective uncertainty in CI users compared to NH participants [51]. However, in order to account for this, we decided to make several back and forth presentations of the same pitch differences between the A and B sounds of the ABA triplets [50], and to measure perception over the entire sequence. Here, we can assume that the subjective uncertainty would be compensated over the time of the sequence. Recent studies have also shown that this decreased streaming in CI users was not only due to an increased decision time, as it depends on electrode separation, but also on tone repetition time [107,108,109], similarly to NH participants [47]. Moreover, even if this kind of perceptual task is rather simple, the performance correlates with speech perception in noise [42,109]. Taken together, these findings suggest that a task using streaming segregation is a simple and rapid way to evaluate the hearing-in-noise abilities of CI users independently of their phonological skills.

As pitch is important for non-verbal auditory cues in speech, we also evaluated the perception of emotional prosody in our participants. Using a simple paradigm of the emotion categorization of short sentences, we demonstrated a deficit of emotion recognition in CI users compared to NH participants. This deficit was particularly pronounced for joyful and sad sentences, which CI users tended to confound more frequently than did NH participants. Interestingly, the same pattern of deficit was found in NH participants with vocoded sentences, with a deficit related to the number of vocoded channels, and also a specific deficit for neutrality. This demonstrates efficiently the poor perception of emotional prosody in CI users that has already been documented before [28,37,38,110], and allows for a better characterization of this deficit depending on the emotion. Moreover, here, the double paradigm using both emotion categorization and intensity ratings allowed us to show that the perception of emotional prosody is not fully disrupted in CI users. The intensity ratings of the CI users and NH participants did not differ. This result reflects the potential capacities of CI users to correctly perceive emotional prosody on an implicit level. Indeed, in order to rate the intensity of an emotion, no conscious representation of a given stimulus was required. The intensity ratings are more linked to lower activation levels of the representation of this stimulus. Thus, the results of the intensity ratings in CI users reflect a potential implicit preservation of pitch processing in the case of emotional prosody [32], as was already demonstrated in another deficit of pitch perception: congenital amusia [76,111,112,113]. Interestingly, the pattern of results was different for NH participants with vocoded sentences. Indeed, these participants showed decreased intensity ratings for joy and sadness with four-channel vocoded sentences. This could be explained by the fact that NH participants are not used to hearing degraded speech, and use preferentially explicit strategies to detect and judge intensity of an emotion. As vocoded sentences could seem a little dehumanized for NH participants, they might have assumed that the emotions were less sincere and intense, especially for joy, which is generally rather intense, and for sadness, which can be confounded with neutral. This is also in line with previous work that demonstrated the limitations of the vocoder strategy to simulate the CI in NH participants, revealing that vocoded sounds did not give the same results as CI regarding music appreciation [104].

In each of the presented tasks, the sounds were presented at equal RMS. Therefore, it cannot be excluded that the CI users might have used cues other than pitch, such as loudness or timbre cues, to perform the tasks. However, as each of these tasks was designed to reflect the capacities of CI users to process pitch-changing stimuli in ecological situations, we chose to only vary one cue (frequency) and not to use roving of intensity in these tasks, which also avoided interference and overly difficult tasks. Note that if CI users were able to use cues other than pitch, this would reduce the between-group differences if anything. For the PCD task, one could argue that loudness could provide sufficient information to perform the task for CI users. However, this remained the only pitch-related task which revealed a deficit for these participants, so loudness cues were not sufficient. For the DCI task, loudness cues were not expected to be correlated to pitch direction cues given the frequency roving across trials, hence these loudness cues were likely to be insufficient to perform the task. In conclusion, we can assume that pitch cues, which were manipulated in our tasks, were indeed used by the CI participants.

Both bimodal and bilateral CI users were recruited in the present study, as this assessment tool was designed to be performed in various populations with hearing deficits. All of the participants retained their regular device settings (CIs or CI plus conventional hearing aid), as we wanted to reproduce natural listening conditions for each participant, to have the reflection of difficulties participants could experience in their everyday life. We acknowledge that the residual hearing of bimodal users could have helped these patients to perform the tasks. However, we suggest that it was not sufficient to overcome all of their deficits, as we still observed reduced performances of CI users compared to NH participants. Moreover, the additional analyses of the performance of bimodal versus bilateral CI users revealed no differences between the two groups. In the future, it would be interesting to specifically test bimodal users with and without their hearing aids in order to ascertain the specific deficit relating to their CI, and to compare their performance with bilateral users, as it has already been shown that their perception can differ [19,31,33,114].

There was an age difference between the two groups of participants (the NH listeners were significantly younger than the CI users). This difference could have played a role in the difference of performance observed in the PCD task for small pitch changes, for the AS test, or for prosody recognition. However, we could argue that this difference of age was not the main factor, as no difference of group was observed for DCI or STM, and no significant correlation was observed between the age of the CI users and their performance. Furthermore, the deficits in the PCD task, the AS task, and the EMO task were elicited in the NH listeners by the vocoding of the sounds. Moreover, even if the cognitive abilities of participants were not impaired in general (there were no diagnosed psychiatric or neurological disorders), we cannot exclude that age-related central auditory processing deficits could have added some deficit in the CI users. Indeed, age-related hearing loss has been shown to have an effect on the auditory networks and the cognitive abilities of participants, such as working memory abilities, attentive functions, and speech perception [46]. In our present study, it is worth pointing out that for some of our tasks, the CI users performed as well as the NH participants, suggesting that the effect of age on auditory central cognitive abilities and the performances tested here might be rather minor (no effect of the age as a covariate in linear regression models was visible in the CI users’ performance). In order to fully dissociate the effect of CIs and the potential effect of age-related cognitive disorders, it would be interesting to test older normal-hearing participants with the tests of the present battery.

Overall, the present listening tests allow for the rapid (30 min) and easy (using a touchscreen tablet) characterization of several facets of non-verbal auditory perception in CI users and NH participants. The results were not found to depend on the time of use of the CI, as no correlation was found between the time of implantation and the performances of CI users (Pearsons’ correlation, all *r* < 0.442, all *p* > 0.2). We suggest, however, that the type of implant, sound processor, or the type of implantation (longer electrodes in particular) could influence the results of participants, as this affects pitch perception [115], which would need to be tested in larger-scale studies. The battery includes basic pitch detection and categorization tasks, but also tasks which are closer to the listening experience of everyday life, such as emotional prosody perception and the challenge of sound source segregation. In the population of CI users tested presently, DCI and STM tasks showed similar pattern of results and did not reveal any deficit. However, as this assessment tool was designed to be used for several pathological populations (and not only CI users with no cognitive deficits), we still recommend performing the entire testing session with the five tasks in order to fully uncover the potential deficits of the tested participants.

### 4.2. Benefit of Audiovisual Cues for Non-Verbal Auditory Tasks

In the present study, we also investigated a potential enhancement of pitch perception by additional visual stimulation. In the PCD task, we used non-task–informative visual cues which provided only temporal cues about the tones (and in particular, their onset and duration). Indeed, these visual cues did not provide the correct answer to perform the task, but rather gave information about the onset of the sounds. As the visual cues were not informative regarding the pitch of the tones, the visual information with its adequate timing might boost participants’ dynamic attendance to the onsets of the tones, as the tones were presented in a regular, isochronous sequence in the PCD task [116,117,118]. We found only weak evidence, if any, for an improvement of performance in audio-visual trials compared to audio-only trials in CI users. However, the trend was in the expected direction, notably based on a previous study in control and amusic participants, which incorporated a larger number of trials [63].

In the STM task, the visual cues were informative for the pitch dimension, but they were present only during the first melody and the retention delay. Hence, they did not inform them about the correct answer (which was the case in the DCI task). For the STM task, we observed a general improvement of the scores with audiovisual trials compared to the auditory trials in both populations. This result demonstrates that informative visual cues can help participants to enhance auditory perception and encoding, leading to better memory recognition performance, even though these cues were not present during the presentation of S2. In the DCI task, the visual cues were fully informative: they were sufficient to carry out the task without listening to the audio. The results revealed an improvement with audiovisual trials compared to auditory trials in both groups, and for NH participants with vocoded sounds. The visual benefit was even more pronounced in the CI users, who even outperformed the NH participants in the auditory trials. Even if the DCI task could be carried out using only visual cues, they were only presented in half of the trials. The purpose of integrating audio-visual trials in the DCI task was not to provide any specific information, but rather for the sake of completeness, in order to obtain visual information on half of the trials for the three pitch-based tasks. The CI users seemed to better integrate multisensory information and benefit more strongly from this integration than did the NH participants [64,66,69]. Indeed, the scores of the NH participants with audiovisual trials were not at the ceiling, suggesting that these participants might still be relying on auditory perception to carry out the task (as they were requested to do) and made less use of the visual information.

In conclusion, the results from these three tasks showed that visual cues could boost the performance of the participants in pitch perception tasks, both for CI users and NH participants. This suggests that even non-informative visual information (as in the PCD task) or partially informative information (as in the STM task) could help the participants to process pitch information. However, it would require more trials and longer sessions to assess these effects more completely, as they were only small effects in the current data set.

Over the past few years, many training and rehabilitation strategies have been developed to enhance the pitch perception abilities in CI users. For instance, some training focused on auditory musical training [34,39,105,119,120,121,122,123,124,125]. Most of these trainings used the Melodic Contour Identification task [102,105], in which a subject hears a melody, and has to recognize its contour and select the correct response among several contour visual representations. While most of these studies looked at the effect of the training of speech perception [119,120,122], some have demonstrated some improvement of intentional [34] or emotional prosody recognition in CI users [39]. The other studies proposed training strategies based on multi-sensorial integration, which was previously demonstrated to be enhanced in CI users [64]. For example, audio-motor integration [126] using electrotactile stimulation showed some minor improvement in speech perception [127]. Based on the previous results in CI users [64,65,66,128] and on the present results of our audiovisual tasks, it seems that audiovisual training could be a good strategy to enhance the auditory perception in CI users [129]. The present results are in favor of a training strategy based on informative cues, as demonstrated in the DCI and STM tasks. These informational visual cues could benefit pitch perception, especially when the difficulty is increased. Nevertheless, a training strategy based on non-informative cues could be enough to allow for multisensory integration and to enhance pitch perception. In the present study, CI users might even have benefited from a small training effect with visual cues [74], as their results in the DCI or STM tasks were similar to those of the NH participants. Indeed, these visual cues could have helped them to understand the meaning of the task (Up or Down for DCI), and could even give them a hearing strategy to perform the STM task, notably based on contour information [130]. In conclusion, these perception data provide some interesting insights for further training strategies in CI users, as well as for other populations with pitch perception deficits (e.g., individuals with congenital amusia).

## 5. Conclusions

Overall, the findings of the present study suggest that the five listening tests of PCD, DCI, STM, AS, and EMO can be used to characterize non-verbal auditory perception in participants with hearing difficulties (here CI users), as well as in NH participants with vocoded sounds. This assessment allows for the rapid (30 min) detection of pitch difficulties with auditory non-verbal sounds, as well as emotional prosody processing and stream segregation capacities, to be used easily in research studies. In order to obtain the full normal value range, it would be interesting to run this battery on a large sample of NH participants with various age and socioeconomic backgrounds in future studies. Moreover, the three pitch tasks using visual cues allowed us to better characterize multisensory integration in NH participants and CI users. These results could be a good starting point to devise a new audiovisual training procedure for participants with pitch perception difficulties [122], notably regarding the types of audiovisual cues to use (informative and non-informative). The five listening tests can then be used to monitor the enhancement of pitch perception abilities in participants (i.e., pre/post-training assessment; Pralus et al., in progress).

## Figures and Tables

**Table 1:** Demographic data of the participants (CI and controls). The standard deviation is indicated in parentheses. The groups were compared with t-tests (two-sided), except for sex, where a Chi2 test was used (Qobs = 3.3). The effect sizes (Cohen’s d) and lower and upper confidence intervals (95%) are reported. CI: cochlear implant; M: male; F: female; R: right-handed; L: Left-handed; NA: not applicable.

Group	CI (6 Unilateral and 4 Bilateral)	Controls (*N* = 10)	*p*-Value (Group Comparison)	Effect Size (Lower and Upper Confidence Interval at 95%)
**Sex**	8M 2F	4M 6F	0.07	
**Age (years)**	51 (±14) Min: 24 Max: 73	22.1(±1.7) Min: 20 Max: 25	<0.001	2.9 (1.6–4.2)
**Education (years)**	16.1 (±2.8) Min: 10 Max: 20	15.5 (±1.2) Min: 14 Max: 17	0.5	0.28 (−0.6–1.2)
**Musical education (years)**	1.5 (±4.7) Min: 0 Max: 15	0.6 (±1.6) Min: 0 Max: 5	0.6	0.26 (−0.6–1.1)
**Laterality**	9R, 1L	9R, 1L	1	
**Right Ear**	8 implants, 2 hearing-aids	NA		
**Left Ear**	6 implants, 4 hearing-aids	NA		
**Unilateral implant (*n* = 6):** **Duration (years)**	2.33 (±1.5) Min: 1 Max: 5	NA		
**Bilateral implants (*n* = 4):** **First implant Duration (years)**	6.75 (±6.4) Min: 2 Max: 16	NA		
**Bilateral implants (*n* = 4):** **Second implant Duration (years)**	5 (±4.5) Min: 1 Max: 11	NA		

**Table 2:** Results of the Bayesian mixed repeated measure ANOVAs for each task (PCD, STM, DCI, AS, EMO), comparing NH participants for four sound types (original, vocoded with 16, eight, and four channels).

	Models	P(M)	P(M|Data)	BF_M_	BF_10_	Error %
**PCD**	Null model (incl. subject)	0.2	0.136	0.629	1.000	
	**Sound Type**	**0.2**	**0.555**	**4.986**	**4.085**	**0.865**
	Sound Type + Modality	0.2	0.164	0.782	1.204	3.341
	Sound Type + Modality + Sound Type × Modality	0.2	0.107	0.479	0.787	3.020
	Modality	0.2	0.039	0.161	0.285	0.956
**DCI**	Null model (incl. subject)	0.2	3.164 × 10^−7^	1.266 × 10^−6^	1.000	
	**Modality**	**0.2**	**0.881**	**29.605**	**2.784 × 10^6^**	**5.315**
	Sound Type + Modality	0.2	0.099	0.438	311,979.244	1.818
	Sound Type + Modality + Sound Type × Modality	0.2	0.020	0.083	64,215.941	1.075
	Sound Type	0.2	3.109 × 10^−8^	1.244 × 10^−7^	0.098	1.218
**STM**	**Null model (incl. subject)**	**0.2**	**0.522**	**4.370**	**1.000**	
	Sound Type	0.2	0.239	1.255	0.458	0.612
	Modality	0.2	0.147	0.687	0.281	1.416
	Sound Type + Modality	0.2	0.071	0.305	0.136	3.380
	Sound Type + Modality + Sound Type × Modality	0.2	0.021	0.088	0.041	1.351
**AS-total time**	Null model (incl. subject)	0.2	2.668 × 10^−49^	1.067 × 10^−48^	1.000	
	**Sound Type + Percept + Sound Type** **× Percept**	**0.2**	**0.875**	**28.028**	**3.280 × 10^48^**	**3.159**
	Percept	0.2	0.116	0.523	4.332 × 10^47^	1.046
	Sound Type + Percept	0.2	0.009	0.038	3.484 × 10^46^	1.184
	Sound Type	0.2	1.883 × 10^−50^	7.532 × 10^−50^	0.071	2.129
**AS-frequency**	Null model (incl. subject)	0.5	0.073	0.079	1.000	
	**Sound Type**	**0.5**	**0.927**	**12.720**	**12.720**	**0.307**
**EMO-recognition**	Null model (incl. subject)	0.2	1.560 × 10^−20^	6.240 × 10^−20^	1.000	
	**Sound Type + Emotion + Sound Type** **× Emotion**	**0.2**	**0.886**	**31.106**	**5.680 × 10^19^**	**0.682**
	Sound Type + Emotion	0.2	0.114	0.514	7.301 × 10^18^	0.934
	Sound Type	0.2	6.305 × 10^−5^	2.522 × 10^−4^	4.042 × 10^15^	0.600
	Emotion	0.2	3.105 × 10^−19^	1.242 × 10^−18^	19.904	0.658
**EMO-intensity**	Null model (incl. subject)	0.2	3.605 × 10^−6^	1.442 × 10^−5^	1.000	
	**Sound Type + Emotion + Sound Type** **× Emotion**	**0.2**	**0.999**	**4038.321**	**277,105.29**	**0.859**
	Sound Type + Emotion	0.2	8.423 × 10^−4^	0.003	233.632	0.864
	Sound Type	0.2	1.304 × 10^−4^	5.218 × 10^−4^	36.182	0.789
	Emotion	0.2	1.320 × 10^−5^	5.280 × 10^−5^	3.661	0.550

The best model for each task is in bold font. P(M): prior probability assigned to the model; P(M|data): probability of the model knowing the data; BF_M_: Bayesian Factor of the model; BF_10_: Bayesian Factor of the model compared to the null model.

**Table 3:** Results of the Bayesian mixed repeated measures ANOVAs for each task (PCD, STM, DCI, AS, EMO), comparing the NH participants and CI users (Group). The best model for each task is in a bold font. P(M): prior probability assigned to the model; P(M|data): probability of the model knowing the data; BF_M_: Bayesian Factor of the model; BF_10_: Bayesian Factor of the model compared to the null model.

	Models	P(M)	P(M|data)	BF_M_	BF_10_	Error %
**PCD**	**Null model (incl. subject)**	**0.2**	**0.284**	**1.586**	**1.000**	
	Modality + Group + Modality × Group	0.2	0.255	1.368	0.897	1.732
	Modality	0.2	0.204	1.026	0.719	1.035
	Group	0.2	0.150	0.706	0.528	0.943
	Modality+ Group	0.2	0.107	0.480	0.377	2.265
**DCI**	Null model (incl. subject)	0.2	1.111 × 10^−4^	4.444 × 10^−4^	1.000	
	**Modality**	**0.2**	**0.464**	**3.465**	**4178.231**	**1.488**
	Modality + Group	0.2	0.301	1.721	2708.463	1.336
	Modality + Group + Modality × Group	0.2	0.235	1.227	2113.544	1.824
	Group	0.2	5.756 × 10^−5^	2.303 × 10^−4^	0.518	0.660
**STM**	Null model (incl. subject)	0.2	0.226	1.170	1.000	
	**Modality**	**0.2**	**0.394**	**2.605**	**1.743**	**0.945**
	Modality + Group	0.2	0.195	0.967	0.860	1.415
	Group	0.2	0.105	0.470	0.465	0.647
	Modality + Group + Modality × Group	0.2	0.079	0.345	0.351	1.767
**AS-total time**	Null model (incl. subject)	0.2	1.367 × 10^−17^	5.469 × 10^−17^	1.000	
	**Perception + Group + Perception × Group**	**0.2**	**0.878**	**28.803**	**6.422 × 10^16^**	**1.531**
	Percept	0.2	0.090	0.394	6.558 × 10^15^	0.953
	Perception + Group	0.2	0.032	0.133	2.360 × 10^15^	1.629
	Group	0.2	4.768 × 10^−18^	1.907 × 10^−17^	0.349	1.044
**AS-frequency**	Null model	0.5	0.163	0.195	1.000	
	**Group**	**0.5**	**0.837**	**5.125**	**5.125**	**7.765 × 10^−4^**
**EMO-recognition**	Null model (incl. subject)	0.2	2.095 × 10^−4^	8.380 × 10^−4^	1.000	
	**Emotion + Group + Emotion** **×** **Group**	**0.2**	**0.860**	**24.653**	**4107.699**	**2.294**
	Emotion + Group	0.2	0.123	0.563	588.756	0.879
	Emotion	0.2	0.014	0.058	67.967	0.347
	Group	0.2	0.002	0.007	8.747	2.277
**EMO-intensity**	Null model (incl. subject)	0.2	0.295	1.673	1.000	
	**Emotion**	**0.2**	**0.315**	**1.838**	**1.067**	**0.787**
	Emotion + Group	0.2	0.174	0.843	0.590	0.956
	Group	0.2	0.161	0.769	0.547	0.868
	Emotion + Group + Emotion × Group	0.2	0.055	0.233	0.187	1.238

## Data Availability

The conditions of our ethics approval do not permit public archiving of anonymized study data. Readers seeking access to the data should contact A. Pralus. Access will be granted to named individuals in accordance with ethical procedures governing the reuse of clinical data, including completion of a formal data sharing agreement and approval of the local ethics committee.

## Supplementary Materials

The following are available online at https://www.mdpi.com/article/10.3390/jcm10102093/s1, Figure S1: Results of the NH participants and CI users in the PCD, DCI, and STM tasks (auditory trials only). Table S1: Information on cochlear implant(s) for bilateral and unilateral CI users. Table S2: Percent of answer types for each intended emotion for EMO task, averaged over the 10 NH participants for each Sound Types (Original, vocoded 16, 8, or 4 channels). Table S3: Percent of answer types for each intended emotion for EMO task, averaged over the 10 participants for each group (NH participants and CI users). Table S4: Individual results of CI users.

## Highlights

- Five listening tests were used to assess non-verbal auditory perception.
- CI users showed deficits in pitch discrimination, emotional prosody, and streaming.
- Similar deficits were observed in NH listeners with vocoded sounds.
- Visual cues can enhance CI users’ performance in pitch perception tasks.
