# Supplementary material for: Rapid Assessment of Non-Verbal Auditory Perception in Normal-Hearing Participants and Cochlear Implant Users
Source: J Clin Med. 2021 May 13;10(10):2093. doi: 10.3390/jcm10102093 (PMC8152499; doi:10.3390/jcm10102093)
Supplement: Supplementary file 1 [file jcm-10-02093-s001.zip › jcm-1176404-proofed-supplementary.pdf]

Table S1: Information on cochlear implant(s) for bilateral and unilateral CI users.

Table S2: Percent of answer types for each intended emotion for EMO task, averaged over the 10 NH participants for each Sound Types (Original, vocoded 16, 8, or 4 channels).

Table S3: Percent of answer types for each intended emotion for EMO task, averaged over the 10 participants for each group (NH participants and CI users).

Table S4: Individual results of CI users.

Figure S1: results of the NH participants and CI users in the PCD, DCI, and STM tasks (auditory trials only).

Table S1. Information on cochlear implant(s) for bilateral and unilateral CI users.

Participant	*Age*	*Sex*	*Implant 1 brand*	*Implant type 1*	*Sound processor 1*	*Side 1*	*Implant 1 Duration*	*Implant 2 brand*	*Implant type 2*	*Sound processor 2*	*Side 2*	*Implant 2 Duration*
Bilateral1	56	M	Advanced Bionics	Hi Res 90k Mid Scala	Naïda	L	16	Advanced Bionics	Hi Res 90k Mid Scala	Naïda	R	11
Bilateral2	73	M	Oticon	Neuro Zti	Neuro 2	L	2	Oticon	Neuro Zti	Neuro 2	R	1
Bilateral3	54	M	Advanced Bionics	Hi Res 90k Mid Scala	Naïda	R	3	Advanced Bionics	Hi Res 90k Mid Scala	Naïda	L	2
Bilateral4	24	M	MedEl	Sonata Ti 100	Sonnet	R	6	Medel	Synchrony Flex 24	Sonnet	L	6
Unilateral1	61	M	MedEl	Synchrony Electrode standard	Sonnet	R	2					
Unilateral2	54	M	Advanced Bionics	Hi Res 90k Mid Scala	Naïda	R	2					
Unilateral3	50	F	MedEl	Sonata Ti 100	Sonnet	L	5					
Unilateral4	53	M	Cochlear	CI522	Kanso	R	1					
Unilateral5	47	M	Cochlear	CI522	CP910	L	3					
Unilateral6	34	F	MedEl	Synchrony Electrode standard	Sonnet	R	1					

Implant durations are in years.

Table S2: Percent of answer types for each intended emotion for EMO task, averaged over the 10 NH participants for each Sound Types (Original, vocoded 16, 8 or 4 channels).

	**Answered / Expected**	***Joy***	***Sadness***	***Anger***	***Fear***	***Neutral***
Original						
	Joy	**97.2**	0	0	0	2.8
	Sadness	0	**94.4**	2.8	2.8	0
	Anger	0	0	**97.2**	2.8	0
	Fear	2.8	16.7	2.8	**75**	2.8
	Neutral	0	2.8	2.8	0	**94.4**
Vocoded 16						
	Joy	**82.5**	7.5	2.5	5	2.5
	Sadness	7.5	**80**	0	7.5	5
	Anger	0	0	**82.5**	15	2.5
	Fear	12.5	5	15	**67.5**	0
	Neutral	0	22.5	5	7.5	**65**
Vocoded 8						
	Joy	**47.5**	7.5	12.5	25	7.5
	Sadness	0	**65**	2.5	15	17.5
	Anger	0	0	**80**	17.5	2.5
	Fear	2.5	10	27.5	**52.5**	7.5
	Neutral	5	22.5	2.5	10	**60**
Vocoded 4						
	Joy	**27.8**	5.6	33.3	19.4	13.9
	Sadness	2.8	**36.1**	22.2	13.9	25
	Anger	2.8	0	**77.8**	11.1	8.3
	Fear	5.6	5.6	33.3	**44.4**	11.1
	Neutral	0	38.9	19.4	16.7	**25**

Correct answers are on the diagonal.

Table S3: Percent of answer types for each intended emotion for EMO task, averaged over the 10 participants for each group (NH participants and CI users).

Correct answers are on the diagonal.

	Answered / Expected	*Joy*	*Sadness*	*Anger*	*Fear*	*Neutral*
NH participants						
	Joy	**97.2**	0	0	0	2.8
	Sadness	0	**94.4**	2.8	2.8	0
	Anger	0	0	**97.2**	2.8	0
	Fear	2.8	16.7	2.8	**75**	2.8
	Neutral	0	2.8	2.8	0	**94.4**
CI users						
	Joy	**57.5**	20	5	15	2.5
	Sadness	7.5	**62.5**	0	5	25
	Anger	2.5	0	**77.5**	12.5	7.5
	Fear	0	7.5	20	**62.5**	10
	Neutral	0	15	0	0	**85**

Table S4: Individual results of CI users.

CI users	PCD_A	PCD_AV	DCI_A	DCI_AV	STM_A	STM_AV	AS_percept	AS_freq	EMO_categ	EMO_int
Bilateral1	98,43	90,41	76,67	100,00	65,83	91,67	**31,20**	**126,88**	75,00	3,21
Bilateral2	82,46	89,36	65,87	100,00	71,61	70,14	**24,33**	**159,46**	**30,00**	**1,50**
Bilateral3	90,29	100	70,74	100,00	Missing data	Missing data	4,17	38,25	**55,00**	2,25
Bilateral4	100,00	95.84	79,91	100,00	91,29	100,00	**33,00**	**146,67**	85,00	2,73
Unilateral1	86,95	83,41	64,92	100,00	83,34	87,50	**28,07**	**127,40**	**50,00**	2,25
Unilateral2	86,93	88,30	89,91	96,67	67,08	100,00	**17,43**	**182,46**	75,00	3,00
Unilateral3	**78,01**	95,57	81,57	100,00	88,89	93,75	**22,21**	**140,59**	**65,00**	4,04
Unilateral4	94,44	100	90,71	100,00	100,00	100,00	7,65	29,75	100,00	3,19
Unilateral5	90.66	90.46	69,79	100,00	93,75	81,25	7,10	29,75	80,00	2,94
Unilateral6	90.43	88.33	72,62	100,00	81,67	66,67	**25,77**	**134,50**	75,00	4,02
NH mean	93,46	86,17	74,33	89,08	82.08	89.46	9,45	49,84	91,67	3,31
cutoff	79.15	64.6	32,93	45,90	46.94	62.38	16,47	124,23	69,31	2,25

For PCD, DCI and STM tasks, results of auditory and audiovisual trials are shown separately. For PCD, DCI, STM and EMO_categ, results are presented in percentage of correct responses. For AS_percept, time in seconds spent in the percept of one stream is indicated. For AS_freq, mean sound frequency of the difference between A and B at percept change is presented. NH participants' mean score is indicated as well as the cutoff corresponding to the mean of NH participants results minus two standard deviations (for AS_percept and AS_freq, cutoff is the mean plus two standard deviation). Individual scores of CI users that fell below the cut-off based on NH data are highlighted in bold.

A: auditory, AV: audiovisual, PCD: Pitch Change Detection, DCI: Direction Change Identification, STM: Short-Term Memory, AS_percept: Auditory Sream segregation, total duration in seconds of the perception of one stream (note that total stimulus length is 80 seconds), AS_freq: Auditory Stream segregation, mean frequency of the B sounds in ABA triplets at change of percept (note that the frequency of A in the ABA triplets is 196 Hz), EMO_categ= mean correct categorization of Emotion, EMO_int= mean intensity ratings of Emotion.

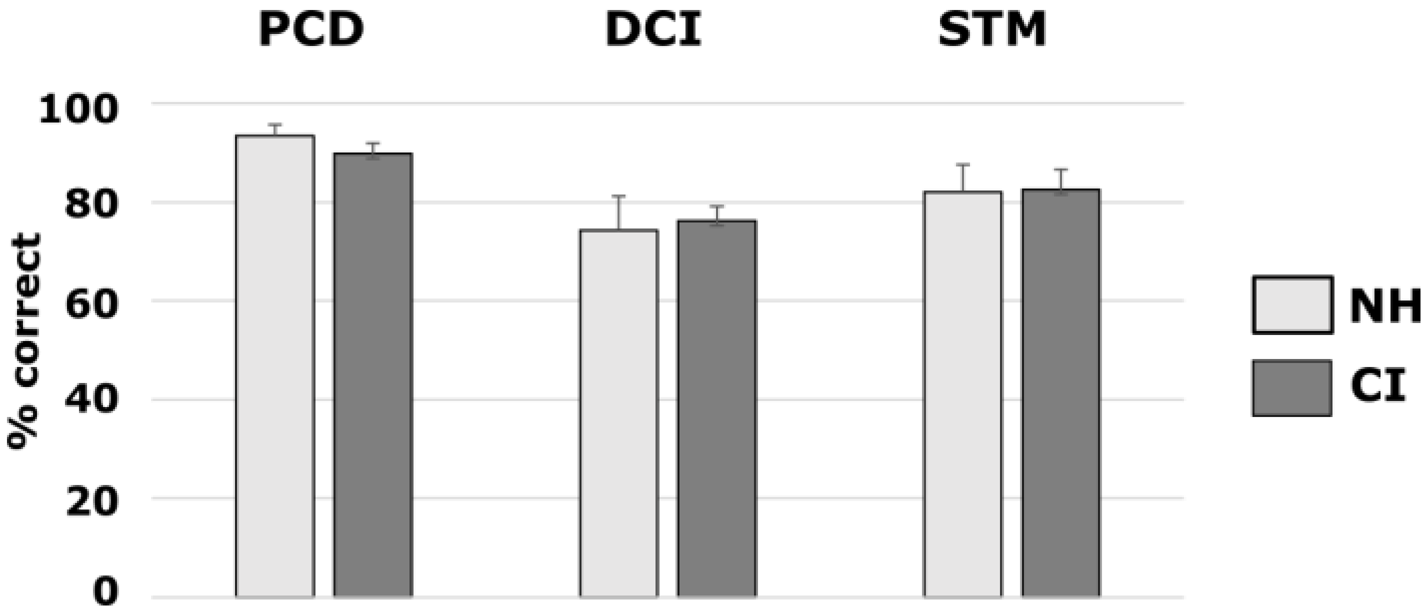

Figure S1: Results of the NH participants and CI users in the PCD, DCI and STM tasks (auditory trials only). *Percentage of correct responses for trials are reported for PCD, DCI, and STM for each group. Error bars represent standard error of the mean.*
